# Occupation and tongue cancer in Nordic countries

**DOI:** 10.1186/s12903-024-04172-2

**Published:** 2024-04-29

**Authors:** Johanna Peltonen, Rayan Nikkilä, Ahmed Al-Samadi, Antti Mäkitie, Jan Ivar Martinsen, Kristina Kjaerheim, Elsebeth Lynge, Par Sparen, Laufey Tryggvadottir, Elisabete Weiderpass, Tuula Salo, Eero Pukkala

**Affiliations:** 1 Department of Oral and Maxillofacial Diseases, Clinicum, Faculty of Medicine, University of Helsinki, Helsinki, Finland; 2 Translational Immunology Research Program (TRIMM), University of Helsinki, Helsinki, Finland; 3 Department of Otorhinolaryngology – Head and Neck Surgery, University of Helsinki, HUS Helsinki University Hospital, Helsinki, Finland; 4 Division of Ear, Nose, and Throat Diseases, Department of Clinical Sciences, Intervention and Technology, Karolinska Institutet and Karolinska Hospital, Stockholm, Sweden; 5 Research Program in Systems Oncology, Faculty of Medicine, University of Helsinki, Helsinki, Finland; 6 The Cancer Registry of Norway at the Norwegian Institute of Public Health, Oslo, Norway; 7 Zealand University Hospital, Nykøbing Falster, University of Copenhagen, Denmark, Copenhagen, Denmark; 8 Department of Medical Epidemiology and Biostatistics, Karolinska Institutet, Stockholm, Sweden; 9 Icelandic Cancer Registry, Reykjavik, Iceland; 10 Faculty of Medicine, University of Iceland, Reykjavik, Iceland; 11 International Agency for Research on Cancer, Lyon, France; 12 Cancer and Translational Medicine Research Unit, University of Oulu, Oulu, Finland; 13 Finnish Cancer Registry, Institute for Statistical and Epidemiological Cancer Research, Helsinki, Finland; 14 Health Sciences Unit, Faculty of Social Sciences, Tampere University, Tampere, Finland; 15 Institute of Dentistry, School of Medicine, Faculty of Health Sciences, University of Eastern Finland , Kuopio, Finland

**Keywords:** Tongue cancer, Nordic, Occupation

## Abstract

**Purpose:**

Almost 200,000 tongue cancers were diagnosed worldwide in 2020. The aim of this study was to describe occupational risk variation in this malignancy.

**Methods:**

The data are based on the Nordic Occupational Cancer (NOCCA) study containing 14.9 million people from the Nordic countries with 9020 tongue cancers diagnosed during 1961–2005. The standardized incidence ratio (SIR) of tongue cancer in each occupational category was calculated using national incidence rates as the reference.

**Results:**

Among men, the incidence was statistically significantly elevated in waiters (SIR 4.36, 95% confidence interval (CI) 3.13-–5.92), beverage workers (SIR 3.42, 95% CI 2.02-5.40), cooks and stewards (SIR 2.55, 95% CI 1.82-3.48), seamen (SIR 1.66, 95% CI 1.36-2.00), journalists (SIR 1.85, 95% CI 1.18-2.75), artistic workers (SIR 2.05, 95% CI 1.54-2.66), hairdressers (SIR 2.17, 95% CI 1.39-3.22), and economically inactive persons (SIR 1.57, 95% CI 1.42-1.73). Among women, the SIR was statistically significantly elevated only in waitresses (SIR 1.39, 95% CI 1.05-1.81). Statistically significant SIRs ≤ 0.63 were observed in male farmers, gardeners, forestry workers and teachers, and in female launderers.

**Conclusions:**

These findings may be related to consumption of alcohol and tobacco, but the effect of carcinogenic exposure from work cannot be excluded.

## Introduction

A total of 378,000 new oral cancer cases (including the lip) were diagnosed worldwide in 2020, 264 000 among men and 114 000 among women [1]. Almost 50% of all oral cancer cases are located in the tongue, and more than 90% of them are squamous cell carcinomas [2, 3]. The 5-year relative survival of the tongue cancer patients diagnosed 2011–2020 in Finland was 64% in men and 75% in women [4]. The main risk factors for oral tongue cancer are smoking and alcohol consumption. Mucosal changes, such as erythroplakia and lichen planus may also increase the risk [5, 6].

The incidence of oral tongue cancer is on the rise among people < 45 years old worldwide, but the reason for the increase is unknown [7]. In the USA, the incidence of tongue cancer increased from 1973 to 2012 among people younger than 50 years, especially among women, although these birth cohorts did not smoke or use alcohol more than previous generations [8]. In the Nordic countries the incidence of tongue cancer has been rising among both sexes during the last decades [9]. Researchers have been trying to identify other risk factors, such as low consumption of fruit and vegetables [10], stress, oral hygiene, and family history [11].

The aim of this study, based on the data of the Nordic Occupational Cancer (NOCCA) project, was to determine the occupational risk variation in tongue cancer [12].

## Materials and methods

The NOCCA cohort includes 14.9 million persons, born in 1896–1960, who participated in any computerized population census in 1960–1990 in the Nordic countries at the age of 30–64 years (2.0 million persons in Denmark, 3.4 million persons in Finland, 0.1 million persons in Iceland, 2.6 million persons in Norway, and 6.8 million persons in Sweden). All Nordic residents are given a personal identity code, and this was used to link data from the registries used in the study. Information on tongue cancer diagnoses (ICD-10 codes C01, C02) was based on the national cancer registries [12]. The follow-up started when the person entered the cohort and ended at emigration, death, or country-specific common closing date (end of year 2003 in Denmark and Norway, 2004 in Iceland, and 2005 in Finland and Sweden). Persons were classified based on the occupation recorded in the first census in which the person participated. The original occupational codes used in national census files were converted to 53 occupational categories and a category of economically inactive persons.

The relative level of tongue cancer incidence for an occupational category is described by the standardized incidence ratio (SIR), with the tongue cancer incidence rates for the entire national study populations used as reference rates. The analysis was stratified by country, sex, 5-year age groups, and 5-year calendar periods. For this study, the stratum-specific results were combined with broader categories of age (30–49, 50–69, 70+).

## Results

Altogether 9020 new tongue cancer cases were diagnosed during the follow-up period (Table 1). Among men, statistically significantly elevated SIRs > 1.50 were found in waiters, beverage workers, cooks and stewards, seamen, journalists, artistic workers, hairdressers, and the economically inactive (Table 2). SIR was > 1.50 also among male dentists, but this finding was not statistically significant. Statistical significantly decreased SIRs < 0.67 were found among male farmers, gardeners, forestry workers, and teachers.

**Table 1:** Study population and years of follow-up in the Nordic countries, and numbers of tongue cancers by country, sex, and age

Country	Study population (million)	Follow-up	Tongue cancers							
			Women				Men			
			Total	30–49 years	50–69 years	70+ years	Total	30–49 years	50–69 years	70+ years
Denmark	2.0	1971–2003	530	28	266	236	873	66	589	218
Finland	3.4	1971–2005	839	111	377	351	1001	188	587	226
Iceland	0.1	1982–2004	17	1	10	6	23	4	13	6
Norway	2.6	1961–2003	668	52	271	345	1201	133	711	357
Sweden	6.8	1961–2005	1539	143	651	745	2329	258	1328	743
Total	14.9		3593	335	1575	1683	5427	649	3228	1550

**Table 2:** Observed number of tongue cancers (Obs) in the five Nordic countries and standardized incidence ratio (SIR) with 95% confidence interval (95% CI), by sex and occupation

Occupation	Men			Women		
	Obs	SIR	95% CI	Obs	SIR	95% CI
Waiters	41	**4.36**	3.13–5.92	55	**1.39**	1.05–1.81
Beverage workers	18	**3.42**	2.02–5.40	< 5		
Cooks and stewards	40	**2.55**	1.82–3.48	39	0.99	0.70–1.35
Hairdressers	24	**2.17**	1.39–3.22	22	1.34	0.84–2.03
Artistic workers	56	**2.05**	1.54–2.66	13	1.74	0.92–2.97
Journalists	24	**1.85**	1.18–2.75	< 5		
Seamen	108	**1.66**	1.36-2.00	< 5		
Dentists	16	1.59	0.91–2.58	6	1.77	0.65–3.85
Economically inactive persons	401	**1.57**	1.42–1.73	1698	0.98	0.93–1.03
Chimney sweeps	6	1.57	0.58–3.41	< 5		
Laboratory assistants	9	1.28	0.58–2.43	5	0.65	0.21–1.52
Printers	58	1.28	0.97–1.66	16	1.64	0.94–2.67
Painters	93	**1.27**	1.02–1.55	< 5		
Shoe and leather workers	21	1.24	0.77–1.90	5	0.60	0.19–1.40
Military personnel	44	1.00	0.72–1.34	< 5		
Engine operators	110	0.94	0.77–1.13	< 5		
Teachers	96	*0.63*	0.51–0.77	123	1.16	0.96–1.39
Domestic assistants	< 5			82	0.84	0.66–1.04
Forestry workers	62	*0.59*	0.45–0.76	< 5		
Farmers	283	*0.51*	0.45–0.58	77	0.89	0.70–1.11
Fishermen	40	0.68	0.49–0.93	< 5		
Launderers	< 5			10	*0.51*	0.24–0.93
Gardeners	82	*0.58*	0.46–0.72	0.86	0.69–1.05	

Significantly elevated SIRs are shown in bold and decreased SIRs in italics

In women, the only statistically significant SIR (1.39, 95% CI 1.05–1.81) was observed among waitresses. Non-significant SIRs > 1.50 were found among dentists, printers, and artistic workers (Table 2). Low incidence with statistical significance was observed only among launderers (SIR 0.51, 95% CI 0.25–0.93).

Among male waiters, elevated SIRs were found in every age group, but the SIR decreased with age (Table 3). Among seamen, the excess decreased towards the older age groups, while the opposite was true for cooks and stewards, and dentists. Female printers had an elevated SIR (2.4, 95% CI 1.1–4.5) in the group 70 + years; this was the only statistically significant age-specific SIR among women.

**Table 3:** Standardized incidence ratio (SIR) with 95% confidence interval (95% CI) for tongue cancer in selected occupations by sex and age. SIRs are not shown when the observed number of cases is <5.

Occupation	Sex	Age at follow-up		
		30–49 years	50–69 years	70 + years
		SIR (95% CI)	SIR (95% CI)	SIR (95% CI)
Waiters	Men Women	**6.0** (2.9–11.1) 1.5 (0.6–3.3)	**4.3** (2.8–6.1) **1.5** (1.0-2.2)	**3.2** (1.2–6.9) 1.2 (0.8–1.9)
Beverage workers	Men		**3.9 **(2.1–6.6)	
Cooks and stewards	Men Women	1.7 (0.6–3.9)	**2.3** (1.4–3.5) 0.9 (0.5–1.5)	**4.2** (2.3–7.2) 1.1 (0.7–1.7)
Hairdressers	Men Women	2.1(0.7–4.9)	**2.0** (1.1–3.4) 1.5 (0.8–2.6)	**2.5** (1.1–4.7) 0.8 (0.3–1.9)
Artistic workers	Men Women	1.9 (0.8–3.7)	**2.1** (1.5–2.9) 2.2 (0.9–4.3)	**2.0** (1.1–3.4)
Journalists	Men	2.3 (0.8–5.5)	1.5 (0.8–2.7)	**2.4** (1.0-4.9)
Seamen	Men	**2.0** (1.1–3.2)	**1.7** (1.3–2.1)	1.4 (0.9–2.1)
Dentists	Men		1.0 (0.4–2.1)	**3.6** (1.7–6.9)
Economically inactive persons	Men Women	**1.6** (1.2-2.0) 1.1 (0.9–1.3)	**1.8** (1.6-2.0) 1.0 (0.9–1.1)	1.1 (0.9–1.4) 1.0 (0.9-1.0)
Printers	Men Women	0.8 (0.3–1.8)	1.2 (0.9–1.7) 1.3 (0.5–2.8)	**1.7** (1.0-2.7) **2.4** (1.1–4.5)
Painters	Men	1.0 (0.4-2.0)	1.3 (1.0-1.7)	1.3 (0.9–1.9)

Significantly elevated SIRs are shown in bold

## Discussion

In this Nordic registry-based study, we compared the risk of tongue cancer in different occupations. In men, statistically significantly elevated SIRs were found among artistic workers, journalists, seamen, cooks and stewards, beverage workers, waiters, hairdressers, and the economically inactive. In women, only waitresses had a significant association with tongue cancer.

In waiters, the SIR was elevated in both sexes in every age group. Waiters may, more often than the population on average, have an irregular lifestyle, and they may need to work during the night. This can lead to unhealthy lifestyle choices, like unhealthy eating habits, excessive alcohol intake and smoking. Waiters use alcohol more than workers in other occupations. Among Swedish restaurant workers alcohol use was almost seven-fold in 2008–2009 [13], and among Norwegian waiters there were heavy drinkers almost three-fold in 1995 [14] compared to the average population. Waiters in the Nordic countries also smoke more than the average population [15, 16]. Waiters in pubs, bars and nightclubs have been heavily exposed to environmental tobacco smoke in the past [17, 18].

Male cooks and stewards had an elevated SIR, mainly in older ages. Cooks and stewards are exposed to cooking oil fumes during working hours. The cooks need to taste the food regularly, which leads to prolonged acid-attack and continuous mechanical irritation, and may further lead to caries and sharp spots that increase the risk of tongue cancer [19, 20]. It is possible that the elevated SIR seen among cooks and stewards aged 70 + years results from the cumulative exposure to prolonged acid attack and continuous mechanical irritation during the cooking career.

Incidence of tongue cancer was increased similarly in both sexes among dentists. The SIR for both sexes combined was 1.64 (95% CI 1.03–2.48). Among male dentists there was no excess of tongue cancer before the age of 70 years, but a highly elevated SIR in ages 70 + years, whereas among female dentists the SIR did not change with age. This finding suggests that – assuming that occupational ones are the same in both genders – there are non-occupational risk factors among male dentists which increase their risk of tongue cancer in higher ages. Hairdressers are regularly exposed to many chemical products, and the International Agency for Research on Cancer (IARC) has classified the exposure mixture in the hairdressing occupation as probably carcinogenic to humans based on elevated the risk cancers of the bladder and lung among hairdressers observed in previous studies [21]. In the present study, male hairdressers had an elevated at least two-fold SIR for tongue cancer in all age categories, but the SIR among female hairdressers decreased towards older age categories. The different findings between male and female hairdressers suggest that there may be other risk factors explaining the elevated SIRs.

In other occupational groups where the SIR was significantly elevated, we can only speculate the reasons. E.g., poor oral health [22, 23] or unhealthy diet, containing less fruit and vegetables [24], which are risk factors for oral cancer, could play a role in the etiology of tongue cancer, too, but we do not have occupation-specific information of such factors.

Strengths of this study include the large number of cancer cases during the follow-up and an accurate registration of tongue cancer diagnoses and occupational codes. However, because we do not have access to individual-level data on tongue cancer risk behavior factors, such as smoking or excessive alcohol consumption, we cannot estimate the role of these factors, factors likely to explain most of the excess risk in some occupational categories. Another limitation of this study is that only information on current occupation was recorded in the first available census instead of life-time occupational history. It has, however, been shown that occupational stability in Nordic countries is so high that the dilution of SIRs because of changes in occupation is relatively small [12].

Cancer located in the base of tongue has been registered as tongue cancer in the NOCCA database, and not as oropharyngeal cancer as it is classified nowadays. The fraction of cancers of the base of tongue in our data forms 10–20% of all cases. Like oropharyngeal squamous cell carcinoma in general, base of tongue cancer is associated with human papillary virus 16 (HPV16) positivity [25–27], while cancer of the mobile tongue is not [28].

In conclusion, it seems that some work environments may include carcinogens that increase the risk of tongue cancer. On the other hand, there is variation between occupations in factors such as alcohol consumption, smoking, and unhealthy diet [24], which may also contribute to occupational variation in tongue cancer risk.

## Data Availability

The data shown in this article are based on the Nordic Occupational Cancer (NOCCA) project. According to the data permissions, data at an individual level had to be deleted after statistical analyses were done and results checked but can be reconstructed by repeating the same registry linkages with permissions of the original data sources. All tabulated data are available from the corresponding author upon reasonable request, and partly (tabular data by country, sex, and occupation) also from the NOCCA website (https://astra.cancer.fi/NOCCA/).
